# Factors Influencing Birth Preparedness in Rapti Municipality of Chitwan, Nepal

**DOI:** 10.1155/2020/7402163

**Published:** 2020-04-21

**Authors:** Kusmita Silwal, Jiwan Kumar Poudyal, Rajani Shah, Sumitra Parajuli, Yubanidhi Basaula, Sabika Munikar, Kanchan Thapa

**Affiliations:** ^1^Shree Medical and Technical College, Bharatpur, Nepal; ^2^Bharatpur Hospital, Bharatpur, Nepal; ^3^Om Health Campus, Chabahil Kathmandu, Nepal; ^4^Safa Sunaulo Nepal, Kathmandu, Nepal

## Abstract

**Introduction:**

Birth preparedness is crucial for health quality of mother and newborn and acts as a strong contributor in mitigating maternal and newborn mortalities. Different factors are predicted to have an influence upon birth preparedness practice. This paper aims at exploring relationship between various factors and birth preparedness practice.

**Methods:**

A cross-sectional study design was used to find out the relationship between various factors and birth preparedness practice. One hundred sixty-five women residing at ward number 1 of Rapti Municipality, Chitwan who delivered in the last twelve months were selected consecutively and interviewed using a semistructured questionnaire. The collected data were analyzed using descriptive and bivariate techniques.

**Results:**

Three quarters (75.2%) of the respondents had better birth preparedness, institutional delivery was 63.0%, antenatal care (ANC) visit as per protocol was about 62.0%, and about 90% of the respondents had received counseling during ANC. Age, religion, family types, education, age at marriage, parity, number of children, knowledge on birth preparedness, knowledge on danger sign, place for ANC and delivery, and decision-makers were found to be statistically significant (*P* value < 0.05) with birth preparedness practice.

**Conclusion:**

Better knowledge on birth preparedness led to a better preparedness status. Age, religion, family type, education of women and partners, parity, and number of children were the factors that influence birth preparedness. Counseling during ANC played a significant role in birth preparedness.

## 1. Introduction

A safe motherhood program aims at reducing maternal mortality and morbidity [1]. The average annual rate of reduction in maternal mortality is 2.9 percent; however, this is less than half of the 6.4 percent annual rate needed to achieve the sustainable development global goal of 70 maternal deaths per 100,000 live births [2]. Birth preparedness and complication readiness (BPCR) is an intervention included by World Health Orgnization (WHO) as an essential element of the antenatal care (ANC) package [3]. The BPCR intervention package based on the WHO IFC framework is effective in building the capacities of women and in engaging men positively in maternal and neonatal health [4]. Birth preparedness includes active preparation and decision-making for childbirth, pregnancy, and postpartum period by pregnant women and their family members [5, 6]. The effective birth preparedness plan (BPP) can positively influence and affect the health of the mother and newborn and the use of health services [5].

BPCR is nationally endorsed as a component of safe motherhood programs in countries like Ethiopia. Though high emphasis is put on improving BPCR among pregnant women, studies show that few women were saving money, identified skilled birth attendant, and were aware of the danger signs during pregnancy [7]. Nineteen percent of pregnant women residing in Cameroon did not make preparations as required by the BPCR plan [8]. Good BPCR practice was found in Thailand with the major factors influencing birth preparedness to be adult pregnancy, being married, high education, being employed, high income, extended family, multiparity, first ANC visit <12 weeks, and average distance to hospital >2 hours [9]. Low compliance of ANC worldwide is considered as a hurdle for maternal and neonatal child health programs. In Nigeria, seventy-one percent of pregnant women are aware of the danger signs [10].

Complications during childbirth and delivery are common. WHO indicates that ninety-eight percent of stillbirth and newborn death occur in low- and middle-income countries: complications of obstetric and labor are responsible for such mishap. The care during the period is important for improving maternal and child health [11]. Nepal aims to reduce the maternal mortality ratio (MMR) to seventy per lakh live birth and neonatal mortality rate (NMR) to less than one per thousand live birth by 2030 [12]. However, Nepal still has poor birth preparedness practices, low institutional delivery, and assistance by a nonskilled practitioner during delivery [13]. It results in various complications during pregnancy, childbirth, and labor that act as major contributors for neonatal and maternal death [13, 14].

Socioeconomic factors influence the birth preparedness status. Marital status, residence, maternal occupation, partner's educational levels, and type of health facility attended by the pregnant women were found to be significantly associated with birth preparedness [15]. Maternal and neonatal deaths can be mitigated by enhancing birth preparedness practices [8]. The present study aims at exploring the relationship between various factors and birth preparedness practices in a community of Chitwan.

## 2. Materials and Methods

### 2.1. Study Area and Period

This study was conducted in ward number 1 of Rapti Municipality, Chitwan district, Nepal, from January 1 to February 1, 2019. It is one of 77 districts of Nepal and is located in the southwestern part of province 3. It covers an area of 2,238.39 (sq. km) and in 2011 had a population of 579,984 (279,087 male and 300,897 female) people. The Rapti Municipality of Chitwan district lies in the eastern part of Chitwan. Rapti Municipality ward number 1 borders with Lothar river in the east, Rapti municipality ward no. 2 in the west, Rapti municipality ward no. 11 in the north, and the Chitwan national park or Rapti river in the south. The area covered by the Rapti Municipality ward no. 1 is 26.33 (sq. km).

### 2.2. Study Design

A cross-sectional study was carried out among the mothers of the ward 1 of Rapti Municipality of Chitwan who delivered in the past 12 months.

### 2.3. Sample Size and Sampling Methods

The sample size was calculated assuming the maximum heterogeneity of population, i.e., unknown prevalence (*P* = 0.5) at 95% confidence interval and 8% margin of errors using the formula for calculation of sample size as below: Where *n* = minimum sample size, *Zα* = 5% level of significance, i.e., 1.96, *p* = prevalence 50%(0.5), and *q* = 1 − *p*; hence, *q* = 1 − 0.5 = 0.5, *d* = maximum tolerable error set at 8%or 0.08.

Thus, substituting the formula:

(1) $$\begin{array}{c} n\;=\left[\left(1.96\;\times 1.96\right)\;\left(0.5\right)\;\times \left(0.5\right)\right]/\left(0.08\right)2=150. \end{array}$$

Considering 10% nonresponse rate, the desired sample size was calculated as *n* = 150 + 15 = 165.

The two-step sampling procedure was used. First, the ward number 1 was selected purposively and then the respondent consecutively until we reached the desired sample size of 165 on the first contact basis with the researcher.

### 2.4. Data Collection

A pretested semistructured questionnaire was used to collect information from respondents. Pretesting of the data collection tool was done among ten percent of the respondents at the adjoining ward of the municipality. Modification on the questionnaire was done as per the findings of pretesting. Face-to-face interview was done for 15 to 20 minutes by visiting the house of the respondents. Recall bias was minimized by involving women who gave birth recently (in the past 12 months). Content validity was maintained through pretesting. The questionnaire was translated into the local language and was retranslated to English to check for consistency. The questionnaire consisted of 49 questions exploring information on sociodemographic status, knowledge and practices on birth preparedness, family and community role in birth preparedness, and health service-related data.

### 2.5. Data Processing and Analysis

The collected data were then checked for completeness and recoded for analysis. All the data were entered into SPSS version 20.0 for analysis. The data were analyzed using a descriptive and bivariate analysis at 95 percent confidence level. The characteristics were stated significant at the chi-squared and Fisher exact tests at *P* < 0.05.

### 2.6. Ethical Consideration

The present study obtained ethical approval from the Institutional Review Committee of Shree Medical and Technical College (Ref# SMTC-IRC-20190107-10). Informed written consent was obtained from the respondents before data collection. Those respondents who were not willing to give information, who were unable to hear/speak and/or mentally ill were excluded in this study. The voluntary participation of the respondents was ensured throughout the study. Confidentiality and privacy were maintained.

### 2.7. Study Variables

#### 2.7.1. Dependent Variable

The dependent variable was birth preparedness.

#### 2.7.2. Independent Variables

Sociodemographic variables include age, education, caste/ethnicity, religion, occupation, family type, parity, sex of recent child, and age at marriage.

Knowledge-related variables include knowledge about BPCR, source of information, obstetric problems/complications, danger signs, reason of not having BP, delivery place, knowledge about ANC, and visit as per protocol.

Roles of family and neighbors include decision-making and support.

Service-related variables include availability of skilled health workers, availability of drug and equipment, adequacy of physical facilities, distance to health facilities, perception to health services, and satisfaction with care.

### 2.8. Operational Definitions

#### 2.8.1. *Birth Preparedness*

Women were considered prepared for birth when at least one of five components of birth preparation, i.e., identifying transport, saving money, identifying health facility, identifying blood donors, and identifying skilled health providers, was practiced. It was further categorized into poor and better preparation [16]. In the present study, there were 6 questions related to preparation. The categorization was done as follows.

1. Poor preparation: when the respondents had less than two birth preparation as mentioned in definition of birth preparedness
2. Better preparation: when the respondent had three or more birth preparation as mentioned in the definition of birth preparedness

#### 2.8.2. Knowledge

It is the total knowledge of danger sign, antenatal care, and preparation for child birth [17]. In the present study, there were total of 10 knowledge-related questionnaires. The knowledge classification was done based on following scoring

- No knowledge: the respondents who did not answer any question correctly
- Poor knowledge: the respondents who gave less than two correct answers
- Better knowledge: the respondents who gave 3 and more answers correctly.

#### 2.8.3. Counseling

It is the delivery of information regarding birth preparedness that health service provider provides during ANC visit [18]. There were altogether 6 counseling-related questionnaires. The following categorization was done as below

- No counseling: if the health personnel did not give any counseling during ANC to the respondent then it was categorized as no counseling
- Poor counseling: if the health personnel gave 2 or less information during ANC to the respondent then it was categorized as poor counseling
- Better counseling: if the health personnel gave 3 or more information during ANC to the respondents then it was categorized into better counseling.

#### 2.8.4. Level of Satisfaction and Perception

It means the respondents' satisfaction and perceived need of health service were provided by the nearest health facilities or by the health personnel.

#### 2.8.5. Available Health Workers

Presence of health workers in health facility was categorized as sometimes, always, and do not know.

#### 2.8.6. Sufficiency of Physical Infrastructure

Sufficient, insufficient, and do not know [19].

## 3. Results

In this study, seventy-seven percent of respondents were of the age group 21 to 35 years (Table 1). There was nearly eight percent of respondents whose age was 20 or less years. The Hindu and Buddhist respondents were forty-seven and forty-one percent respectively. Nearly seventy-eight percent were Janajati. Thirty-eight percent were educated up to the primary level whereas thirty-four percent had secondary and higher level of education. Similarly, forty-nine percent of the husbands had a primary level education, whereas eighteen percent did not have any formal schooling. Among total respondents, ninety-three percent were housewives or involved in agricultural works, whereas seven percent were engaged in other professions. Agriculture was a major profession among approximately forty-one percent of husbands. The age at marriage was less than 20 years for almost sixty-seven percent of the respondents. At present, fifty-seven percent of the respondents had male children followed by forty-three percent having a female child.

Among the respondents, eighty-four percent had ANC check-up. Among them, only sixty-two percent had four visits as per protocol. The major causes of not visiting for ANC were distance (31%) and lack of information (58%). Similarly, among those who attended ANC, only about fifty-three percent received better counseling. Furthermore, seventy percent of the respondents had better knowledge about danger signs requiring treatment during pregnancy. Seventy-seven percent of the respondents had heard about birth preparedness. The most important source of information reported was health workers (87%) followed by friends/relatives (76%). Furthermore, seventy-five percent reported to have done birth preparation. Among those who did not do any preparation, forty-nine percent reported they did not need to prepare, whereas twenty-nine percent reported they lacked the information about BPP. Seven percent of them also reported having birth preparedness being bad for the child (Table 2).

Sixty-two percent of the respondents reported joint decision-making by husband and wife, and thirty percent of them stated being able to decide themselves about childbirth. Ninety-four percent knew about the place for delivery. However, still, there were thirty-seven percent respondents who gave birth at home. Non-health worker conducted delivery at home. Among the total respondents, forty-three percent had some sorts of complications during pregnancy and delivery. The complications prevalent among the respondents were severe headache (1.4%), vaginal bleeding/severe abdominal pain (53.5%), blurring vision (1.4%), swollen hands and feet (14.1%), and labor lasting more than twelve hours (29.6%) (Table 3).

BPP was further compared with different variables of interest (Table 4). The age factor played an important role in birth preparedness. Seventy-seven percent of the respondents who were between 21 to 35 years reported having better preparedness. Buddhist and Hindu respondents had better preparedness status, whereas Christian respondents had poor preparedness. Among respondents living in nuclear family, 78 percent had better preparedness. Furthermore, among educated respondents eighty percent reported having better preparedness. Similarly, husband's education played a significant role for BPP, and it is found that of the husbands with some sorts of education, 78 percent reported well prepared. Respondents who were married after the age of twenty had better preparedness. Those who had given only one birth were better prepared than others. Similarly, those who had three and more children were least prepared. Relationship of age, religion, family type, education, husband's education, age at marriage, parity, and number of living children was statistically significant (*P* < 0.05) with birth preparedness practice. However, occupation had no significant relationship with birth preparedness (*P* value = 0.512).

Ninety-four percent of the respondents with better knowledge of birth preparedness had better status on BPP. About eighty-six percent who were able to demonstrate better knowledge about danger signs in pregnancy reported to have a better birth preparedness status. Ninety-five percent of the respondents having ANC showed a better preparedness status. Those who were counseled at the time of ANC showed a better preparedness status. Ninety percent of respondents who made the decision about their childbirth along with their husbands were well prepared than others (Table 5).

## 4. Discussion

Birth preparedness has been globally recognized as an essential component of safe motherhood helpful in reducing the delay to reach, seek, and receive care. These delays are avoidable and are crucial in saving the life of the mother and newborn [5]. Decision-making is one of the factors for timely treatment and receiving care. The autonomy of the mother and the decision-making process has a significant contribution in reaching care on time. A study reports that the process is influenced by various other factors such as women education, belief system of family, resource scarcities and financial constraints [20].

A study from Nepal highlighted that men are the major decision-makers in the family [21]. In this study, too, respondents who made the decision jointly with their husbands are found to have better birth preparedness. It is crucial to state the findings from Bangladesh that it is important to engage husband positively in maternal and newborn health [4]. These engagements might also have cultural influences. It is realized about the importance of engaging males in decision-making about birth preparedness from the study of the northeast part of Ethiopia which recommended advocating policies, strategies that can improve awareness and engagement of men in maternal care [22].

The majority of respondents in the present study were of the age group 20 to 35 years which is similar to the study conducted in Western Ethiopia where the mean was 28 (±5) years [23]. The observed differences and similarities might possibly be due to similar sociodemographic characteristics of countries. These factors may influence on birth preparedness. Another study indicates that those mothers aged more than thirty-five years and without education should be given more emphasis on explaining about complication readiness and birth preparedness [24]. Despite of higher frequency of lower-aged mother in this study, similar intervention on BPP seems essential.

In this study, four among ten had only a primary level of education whereas one among ten had completed secondary or higher education. So, important emphasis on education level is required. Similarly, birth preparedness practice was found better among those with higher education than with lower education in Thailand, too [25]. Findings from a narrative review from Nepal advocated for the education linked with job opportunities in order to improve the health status of women [26].

In this study, association between parity and birth preparedness practice was identified. Similarly, as per the study conducted in Thailand, primipara were more likely to prepare themselves for birth and its complications [23]. Likewise, respondents who had attended the antenatal clinic during pregnancy of recent delivery were two times more likely to prepare themselves than those who had not [23]. In Nepal, it is recommended to have at least four ANC visits from skilled service providers [27]. ANC visits are the primary contact visit with health care provider where they receive knowledge and information on birth preparedness. So, this paper gave emphasis on ANC visit and counseling. Thus, the ANC visit can lead to better birth preparedness status through better counseling. More than eight among ten have their first ANC visit; however, only six among ten completed their ≥4 ANC, which is similar to the context in Thailand where seven out of ten pregnant women planned to attend at least four antenatal visits with a skilled provider [25]. The major barriers for ANC visits identified in this study were distance and lack of information about birth preparedness. One among two received better counseling regarding childbirth and pregnancy at the time of ANC visit. Moreover, seven among ten were aware, that if there was any unexpected danger sign, they need to visit the health service provider.

The major source of information about birth preparedness identified in this study was health workers followed by friends/relatives. The health service providers are the valued sources of information for birth preparedness. It is also advocated for the inclusion of messages about birth preparedness as an important package in promoting the institutional delivery in Ethiopia [28]. In this study, eight out of ten women heard about the birth preparedness; however, seven out of ten women were better prepared on BP which is in contrast to the findings of Western Ethiopia, where seven out of ten reported to have known about BP; however, only three out of ten were well prepared for birth and its complications [23]. The findings of this study, however, are similar to that of Thailand where eight out of ten had good BPCR [25]. In this study, the major reasons for poor birth preparedness were lack of information and misconception of birth preparedness being bad for a child.

Birth preparedness is directly focused on reducing newborn death where Nepal has experience of implementing the community-based newborn program [18]. Furthermore, there are still challenges such as inadequate policy environment, resources, funding gaps, inadequate programs, and insufficient supplies and commodities in the health system of Nepal [29].

The immediate family members and relatives were the major birth attendant for home delivery in this study. Similar findings have been presented by another study of Nepal which indicates that about sixty-five percent of home deliveries were being conducted by family members/relatives [30]. Another study reveals that the reason for home delivery is an easy and convenient environment which is also influenced by different sociodemographic characteristics [31]. A published review stated that higher socioeconomic status, education, privileged ethnicities, Hindu people, having access to transport and health services, getting family support, able to decide themselves, empowered, and having a birth preparedness plan are the major determinants of maternal health [26]. Another study concluded that emphasis should be given to women without education, to improve accessibility and provide advice on birth preparedness and danger signs to reduce home delivery [32].

Thus, the present study only provided the status of birth preparedness through cross-sectional study in a ward of Rapti Municipality located at Chitwan of Nepal in 2019. However, there are other information to explore, and still, there are several shortcomings in this paper. Therefore, recommendation on another study with large sample size and a more sophisticated and reliable technique converging the larger areas had been done.

## 5. Conclusions

Better knowledge on birth preparedness led to a better preparedness status. Age, religion, family type, education of women and partners, parity, and number of children were the factors that influenced birth preparedness. Counseling during ANC played a significant role in birth preparedness. Therefore, emphasis should be on counseling through completed ANC about birth preparedness and danger signs.

## Figures and Tables

**Table 1:** Distribution of respondents by sociodemographic characteristics (*n* = 165).

Background characteristics	Number (*n*)	Percent (%)
Age (years)		
≤20	13	7.9
21-35	127	77.0
≥36	25	15.1
Religion		
Hindu	77	46.7
Buddhists	68	41.2
Christian	20	12.1
Family type		
Nuclear	104	63.0
Joint/extended	61	37.0
Caste		
Brahman/Chhetri	45	27.3
Janajati	112	67.9
Dalit	8	4.8
Education		
No schooling	46	27.9
Primary level	63	38.2
Secondary and above levels	56	33.9
Husband's education		
No schooling	30	18.2
Primary level	81	49.1
Secondary and above levels	54	32.7
Occupation		
House wife/agriculture	154	93.3
Working women	11	6.7
Husband's occupation		
Farming	67	40.6
Business	10	6.1
Service	25	15.2
Labor	34	20.6
Foreign employment	29	17.6
Age at marriage (years)		
>20	110	66.7
20 to 34	55	33.3
Sex of recent child		
Male	94	57.0
Female	71	43.0

**Table 2:** Distribution of status of birth preparedness-related practices, knowledge, and status among respondents (*n* = 165).

Characteristics	Number (*n*)	Percent (%)
ANC check-up in last pregnancy (*n* = 165)	139	84.2
ANC visit as per protocol (*n* = 139)	86	61.9
Reason for not going ANC visit (*n* = 26)		
Distance to the health facility	8	30.8
Lack of information	15	57.7
Shyness	3	11.5
Counseling during ANC (*n* = 139)		
No counseling	14	10.1
Poor counseling	52	37.4
Better counseling	73	52.5
Knowledge about danger signs requiring treatment (*n* = 165)		
No knowledge	15	9.1
Poor knowledge	34	20.6
Better knowledge	116	70.3
Heard about birth preparedness (*n* = 165)	127	77.0
Source of information for birth preparedness (*n* = 127)^∗^		
Health worker	110	86.6
Radio/TV	11	8.7
Newspaper	4	3.1
Poster/pamphlet	4	3.1
Friends/relatives	97	76.4
Had birth preparedness (*n* = 165)	124	75.2
Reason of not having birth preparedness (*n* = 41)		
Bad for child	3	7.3
No need to prepare	20	48.8
Lack of information	12	29.3
No problem arises before	6	14.6

^∗^Multiple answers.

**Table 3:** Experiences, knowledge, and satisfaction with service provider during delivery by respondents (*n* = 165).

Characteristics	Number (*n*)	Percent (%)
Final decision-maker (*n* = 165)		
Self	49	29.7
Jointly	102	61.8
Family members/relatives	14	8.5
Know about place for delivery (*n* = 165)	155	93.9
Delivery place (*n* = 165)		
Home	61	37.0
Health institution	104	63.0
Attendant during home delivery(*n* = 61)		
Health workers	2	3.2
Non-health workers	61	96.8
Had complications during pregnancy and delivery	71	43.0
Types of complications (*n* = 71)		
Severe headache	1	1.4
Vaginal bleeding/severe abdominal pain	38	53.5
Blurring vision	1	1.4
Swollen hands and feet	10	14.1
Labor lasting more than 12 hours	21	29.6
Availability of health workers at nearest health facility (*n* = 165)		
Sometimes	72	43.6
Always	73	44.2
Do not know	20	12.1
Perception of services in a health facility (*n* = 165)		
Good	66	40
Average	80	48.5
Do not know	19	11.5
Availability of drugs and equipment (*n* = 165)		
Sometimes	69	41.8
Always	74	44.8
No	2	1.2
Do not know	20	12.1
Sufficiency of physical infrastructure (*n* = 165)		
Sufficient	84	50.9
Insufficient	62	37.6
Do not know	19	11.5
Satisfaction with care and respect provided (*n* = 165)		
Often satisfactory	54	32.7
Satisfactory	90	54.6
Not satisfied/do not know	21	12.7

**Table 4:** Comparison between sociodemographic characteristics with birth preparation practices (*n* = 165).

Characteristics	Birth preparation practice		*P* value
	Poor preparation (*n* (%))	Better preparation (*n* (%))	
Age (years)			
≤20	5 (38.5)	8 (61.5)	0.003^∗^
21-35	29 (22.8)	98 (77.2)	
≥36	14 (56.0)	11 (44.0)	
Religion			
Hindu	13 (16.9)	64 (83.1)	<0.001^∗^
Buddhists	22 (32.4)	46 (67.6)	
Christian	13 (65.0)	7 (35.0)	
Family type			
Nuclear	23 (22.1)	81 (77.9)	<0.009^∗^
Joint/extended	25 (41.4)	36 (58.6)	
Education			
Uneducated	24 (52.2)	22 (47.8)	<0.001^∗^
Educated	24 (20.16)	95 (79.83)	
Husband's education			
Uneducated	18 (60.0)	12 (40.0)	<0.001^∗^
Educated	30 (22.2)	105 (77.7)	
Occupation			
Housewife/agriculture	46 (29.9)	108 (70.1)	0.512^∗∗^
Working women	2 (18.2)	9 (81.8)	
Age at marriage (years)			
<20	45 (40.9)	65 (59.1)	<0.001^∗^
≥20	3 (5.5)	52 (94.5)	
Parity			
Single	8 (14.5)	47 (85.5)	<0.001^∗^
Twice	13 (21.3)	48 (78.7)	
3 times	11 (44.0)	14 (56.0)	
4 and more times	16 (66.7)	8 (33.3)	
No. of children			
Single	8 (13.8)	50 (86.2)	0.011^∗^
Two	14 (22.6)	48 (77.4)	
Three to four	11 (44.0)	14 (56.0)	

^∗^Chi-squared test, ^∗∗^Fisher's exact test.

**Table 5:** Comparison between knowledge, ANC, and decision-makers on the delivery place with birth preparedness practices (*n* = 165).

Characteristics	Birth preparation practice		*P* value
	Poor preparation (*n* (%))	Better preparation (*n* (%))	
Knowledge of birth preparation			
Poor knowledge	4 (30.8)	9 (69.2)	0.015^∗∗^
Better knowledge	7 (6.1)	107 (93.9)	
Knowledge of danger signs for requiring check-up in pregnancy			
No knowledge	14 (93.3)	1 (6.7)	<0.001^∗^
Poor knowledge	17 (50.0)	17 (50.0)	
Better knowledge	17 (14.7)	99 (85.3)	
Knowledge of danger sign during delivery			
No knowledge	7 (87.5)	1 (12.5)	<0.001^∗^
Poor knowledge	25 (56.8)	19 (43.2)	
Better knowledge	16 (14.2)	97 (85.8)	
ANC by protocol			
Yes	4 (4.7)	82 (95.3)	<0.001^∗^
No	19 (35.8)	34 (64.2)	
Counseling during ANC check-up			
No counseling	9 (64.3)	5 (35.7)	<0.001^∗^
Counseling	14 (11.2)	111 (88.8)	
Delivery place			
Home	40 (65.6)	21 (34.4)	<0.001^∗^
Institution	8 (7.7)	96 (92.3)	
Final decision-makers			
Self	29 (59.2)	20 (40.8)	<0.001^∗^
Jointly	10 (9.8)	92 (90.2)	
Family members/relatives	9 (64.3)	5 (35.7)	

^∗^Chi-squared test, ^∗∗^Fisher's exact test.

## Data Availability

The data used to support the findings of this study is available from the corresponding author upon request.
